# Noise Smoothing for Structural Vibration Test Signals Using an Improved Wavelet Thresholding Technique

**DOI:** 10.3390/s120811205

**Published:** 2012-08-10

**Authors:** Ting-Hua Yi, Hong-Nan Li, Xiao-Yan Zhao

**Affiliations:** 1 Faculty of Infrastructure Engineering, State Key Laboratory of Coastal and Offshore Engineering, Dalian University of Technology, Dalian 116023, China; E-Mail: hnli@dlut.edu.cn; 2 Shanghai Municipal Engineering Design Institute (group) Co. Ltd., Shanghai 200092, China; E-Mail: iccee@dlut.edu.cn

**Keywords:** vibration testing, wavelet transform (WT), denoise, wavelet thresholding, sigmoid function

## Abstract

In structural vibration tests, one of the main factors which disturb the reliability and accuracy of the results are the noise signals encountered. To overcome this deficiency, this paper presents a discrete wavelet transform (DWT) approach to denoise the measured signals. The denoising performance of DWT is discussed by several processing parameters, including the type of wavelet, decomposition level, thresholding method, and threshold selection rules. To overcome the disadvantages of the traditional hard- and soft-thresholding methods, an improved thresholding technique called the sigmoid function-based thresholding scheme is presented. The procedure is validated by using four benchmarks signals with three degrees of degradation as well as a real measured signal obtained from a three-story reinforced concrete scale model shaking table experiment. The performance of the proposed method is evaluated by computing the signal-to-noise ratio (SNR) and the root-mean-square error (RMSE) after denoising. Results reveal that the proposed method offers superior performance than the traditional methods no matter whether the signals have heavy or light noises embedded.

## 1. Introduction

Vibration-based structural damage detection methods have attracted considerable attention in recent years for the assessment of health and safety of large civil structures [1]. However, the measured signals are often contaminated by noise originating from both the measurement system and the specimens. The noise embedded in useful signals, sometimes even very heavy, disturbs the reliability and accuracy of measurements, and thus places a fundamental limit on the detection of small defects. Therefore, noise reduction should be treated as an essential processing feature in structural dynamics tests.

Up to now, a large number of signal denoising algorithms have been proposed in the literature, such as the linear low-pass filter [2], the Kalman filter [3], the median filter [4] and adaptive filtering based on neural networks [5]. However, these conventional methods all have inherent failings; for example, a linear low-pass filter is not good in a case whose signal overlaps the noise often in many frequency bands [6]. A moving average and median filter provides no means for detecting noise or filtering locally. Besides, a parametric estimation would be unworkable either due to lack of an *a priori* model or because parametric modeling would constrain the analysis in an undesirable way [7]. Hence, single-scale representations of signals, either in time or in frequency, are often inadequate when attempting to separate signals from noisy data. By combining the time domain and the classical frequency domain analysis, the wavelet transform (WT) becomes a potential tool for data analysis. The increasing interest in this method is due to several factors: (1) simplicity of the approach, (2) reduced computational complexity associated with the algorithm, and (3) the ability to provide simultaneously spectral representation and temporal order of the signal decomposition components. Since Donoho and Johnstone [8,9] originally proposed a method known as the wavelet transform (WT) shrinkage (thresholding) to estimate an unknown smoothed signal from data with noise, the WT has rapidly become very popular for signal denoising. However, the denoising performance of the wavelet shrinkage method is conditioned by several processing parameters, including the type of wavelet, decomposition level, thresholding method, and threshold selection rules. Among these parameters, the key one is the thresholding method. The traditional hard thresholding exhibits some discontinuities and may be unstable or more sensitive to small changes in the data, while in soft thresholding the wavelet coefficients are reduced by a quantity equal to the threshold value which will induce the deviation when the filtered wavelet coefficients is reconstructed by the inverse WT (IWT). To overcome the disadvantages of the hard- and soft-thresholding methods, researchers have proposed lots of improved thresholding methods. Gao [10] presented a kind of non-negative garrote shrinkage method, in which finite sample simulations proved that it generally had smaller mean square error and less sensitivity to slight disturbances in the data than both soft and hard shrinkage. Yoon and Vaidyanathan [11] demonstrated a custom thresholding function which could improve the denoised results significantly. In addition, Song and Zhao [12] proposed three models of threshold estimator, that were the polynomial interpolating thresholding method, compromising method of hard- and soft-thresholding and modulus square thresholding method, and experimental results showed that the improved techniques were efficient.

This paper is concerned with the rational selection of processing parameters in the wavelet based filtering method, and is especially relevant to a novel thresholding method, which generalizes the hard- and soft-shrinkage proposed by Donoho and Johnstone [8]. The remainder of this paper is organized as follows: Section 2 summarizes the basic theory of wavelet denoising. Section 3 describes the proposed sigmoid function based thresholding scheme, as well as how to select the rational wavelet decomposition level and threshold. Section 4 analyzes the discriminations between the proposed and other existing method according to the numerical simulation results. In Section 5, results from the analysis of real measured signals are presented. Finally, conclusions are given in Section 6.

## 2. Mathematical Background

### 2.1. Discrete Wavelet Transform

Considering a signal *x_n_* (n = 1,2,…,*N*) ∈ *L*^2^(*R*), the discrete wavelet transform (DWT) approach represents the time record *x_n_* using a linear combination of basis functions *φ_j,k_* and *ψ_jk_*:

(1) $$x(t)=\sum_{K}s_{J,k}\phi_{J,k}(t)+\sum_{K}d_{J,k}\psi_{J,k}(t)+\sum_{K}d_{J-1,k}\psi_{J-1,k}(t)+\cdots +\sum_{K}d_{1,k}\phi_{1,k}(t)$$

where *s_J,k_*, *d_J,k_*,…,*d_l,k_* are the wavelet coefficients; *J* is a small natural number which depends mainly on *N* and the basis function; and *k* ranges from 1 to the number of coefficients in the specified component.

The basis for the above decomposition is formed from the mother wavelet *ψ*(*t*) and father wavelet *φ*(*t*) by translating in time and dilating in scale:

(2) $$\begin{array}{cc} \Psi_{\text{j},\text{k}}(t)=2^{-j/2}\Psi (2^{-j}t-k) & j,k\in Z \end{array}$$

(3) $$\begin{array}{cc} \phi_{\text{j},\text{k}}(t)=2^{-j/2}\phi (2^{-j}t-k) & j,k\in Z \end{array}$$

where *k* = 1,2,…, *N*/*2*, in which *N* is the number of data record; *j* = 1,2,…, *J*, in which *J* is a small natural number; *Z* is the set of integers.

**Figure 1.**
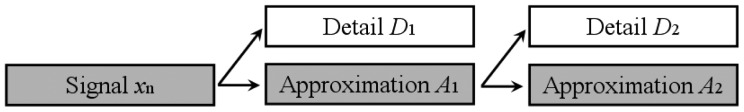
A two-level wavelet decomposition of signal.

Broadly speaking, the DWT performs a recursive decomposition of the lower frequency band obtained from a previous decomposition. As a result, a hierarchical set of approximations and details can be obtained through the various decomposition levels. This procedure, known as the multi-resolution analysis (MRA), was introduced by Mallat and it can be carried out using a computationally efficient algorithm [13]. Figure 1 shows an example of a two level wavelet decomposition of a signal *x_n_*. First, the signal *x_n_* is split into an approximation *A*_1_ and a detail *D*_1_. Then the approximation *A*_1_ is also split into a second level approximation *A*_2_ and *D*_2_.

### 2.2. General Denoise Procedure via Wavelet

Suppose the measured signal *y*(*t_i_*) can be expressed as the sum of two components:

(4) $$y(t_{i})=x(t_{i})+e_{i}$$

where *x*(*t_i_*) is the signal of the detected object buried in the noise *e_i_*. The signal *y*(*t_i_*) is corrupted by noise, in which, much valuable information for structural dynamic characteristics is weakened, and some even disappears, especially in heavy noise. Removing noise completely is impossible. The goal of denoising is to obtain a signal *y*(*t_i_*) as close as possible to *x*(*t_i_*), thus minimizing the effect of *e_i_*.

In general, the wavelet based denoising procedure can be described as follows:

Step (1) Decomposition of the input noisy signal into several levels of approximations and detailed coefficients, using the selected wavelet basis;
Step (2) Thresholding of coefficients. Extract the coefficients actually containing the true signal and discard the others;
Step (3) Reconstruction of the signal using approximations and detailed coefficients by means of the inverse wavelet transform.

The methodology can be applied to the DWT and stationary wavelet transform (SWT), as well as to the wavelet packet transform (WPT). In the present paper, we only focus on the DWT.

## 3. Improved Noise Cancellation Technique

### 3.1. Wavelet Basis Selection

The choice of the mother wavelet for the aforementioned denoising procedure is theoretically arbitrary, but it is critical and important in practice, because it affects the performance of the technique. The properly selected wavelet can give rise to an orthogonal/non-orthogonal analysis; allow a fast algorithm, existence of discrete transforms, possibility of perfect reconstruction, good localization in time and/or in frequency, *etc*. In fact, the coefficients of the wavelet transform represent how well the signal matches the scaled and translated replicas of the mother wavelet. Therefore, it is better to choose a mother wavelet that is as “similar” as possible to the measured signal.

**Figure 2.**
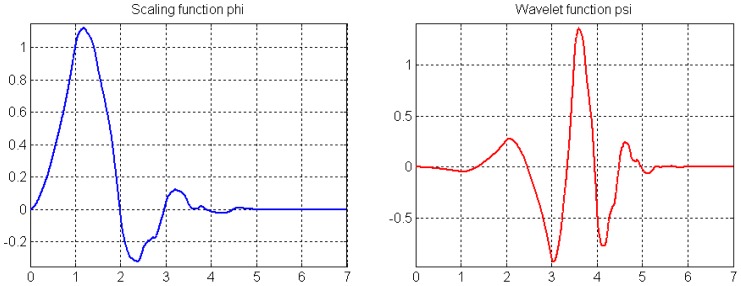
Sketch map of the db4 wavelet.

Since a quantitative criterion for selecting the attenuation factor is not available, in this paper we restricted ourselves to the Daubechies wavelet by trial and error. In all our experiments listed below, the Daubechies 4 (db4) wavelet was found to perform better in preserving fine signal details. As shown in Figure 2, the compact spatial support of db4 wavelet with four vanishing moments can provide better frequency localization and approximation and hence lead to good performance.

### 3.2. Determination of Decomposition Level

The second factor that strongly influences the signal denoising is the level of DWT decomposition. If the level of decomposition is not enough, the improvement of the signal to noise ratio (SNR) will be limited. On the other hand, if the decomposition level is too much, the amount of computation will increase largely, and the noise reduction may be not satisfactory as well. No perfect procedure for choosing the best level of DWT decomposition has yet been proposed, here, a kind of “white noise test” is adopted (Zhang *et al*. [14]).

As stated before, in the DWT a measured signal is broken down into two sub-signals, a detail and an approximate, then the detail sub-signal in turn is broken down into two other sub-signals, respectively, forming a binary tree. Since the amplitude and arrival time of every noise peak is random, this will cause a disturbance that affects the signal or masks the objective signal completely (*i.e.*, the noise in the experimental signal is distributed uniformly over all detail wavelet coefficients with small amplitudes, which generally obeys the white noise distribution). From WT theory, the white noise is still the white noise through the orthogonal WT. But, different with the noise, the desired signal, with certain time and frequency localization, is concentrated into just a few coefficients, which present the non-white noise characteristics. Thus, based on statistical properties of each detail wavelet coefficients, the rational decomposition level could be determined. The procedure for choosing the rational level of DWT decomposition based on “white noise test” is summarized as follows:

Step (1) Decomposition of the input noisy signal into *i* = 1 level of the approximation *A*_1_ and detailed coefficient *D*_1_;
Step (2) Retain the approximation coefficient *A*_1_ obtained by Step (1) and perform the white noise test on the detailed coefficient *D*_1_. Continue to take the decomposition of *A*_1_ if *D*_1_ passes the white noise test;
Step (3) Repeat the above steps, *i.e.*, to perform the white noise test on the corresponding detailed coefficient after the decomposition of each layer until the detailed coefficient cannot pass the white noise test;
Step (4) Abandon the last decomposition result in which the detailed coefficient hasn't passed the white noise test, *i.e.*, the decomposition level should be *n* − 1 if it is decomposed *n* times.

### 3.3. Sigmoid Function Based Thresholding Scheme

The purpose of the thresholding procedure is to eliminate or suppress small value wavelet coefficients which mainly represent the noise content. This process can be carried out following two basic rules: namely the ‘keep-or-kill’ hard thresholding and ‘shrink-or-kill’ soft thresholding introduced by Donoho and Johnstone [8,9]. In hard thresholding, coefficients with absolute values lower than the threshold are set to zero, while soft thresholding in addition shrinks the remaining nonzero coefficients toward zero.

The hard thresholding method is defined as follows:

(5) $${\tilde{\omega }}_{k,j}=\{\begin{array}{cc} \omega_{k,j}, & \text{for}(\left|\omega_{k,j}\right|\geq T)\\ 0 & \text{otherwise} \end{array}$$

Where ω*˜_k,j_* and *ω_k,j_* represent the shrunk and original DWT coefficients, respectively, and *T* denotes the threshold.

The other is called the soft thresholding:

(6) $${\tilde{\omega }}_{k,j}=\{\begin{array}{cc} \mathrm{sgn}(\omega_{k,j})(\left|\omega_{k,j}\right|-T), & \text{for}\left|\omega_{k,j}\right|\geq T\\ 0, & \text{otherwise} \end{array}$$

where sgn(·) is the Signum function, which returns 1 if the element is greater than 0, 0 if it equals zero and −1 if it less than 0. The recovered signal can be obtained from ω*˜_k,j_* by the inverse WT (IWT).

Ideal thresholding functions retain or shrink only wavelet coefficients exceeding a threshold value *T*. However, comparing both hard- and soft-shrinking schemes, it can be seen that the hard thresholding exhibits some discontinuities at ±*T* and may be unstable or more sensitive to small changes in the data. On the other hand, in soft thresholding the wavelet coefficients are reduced by a quantity equal to the threshold value which will induce the deviation when the filtered signal is reconstructed by the inverse WT. To overcome this shortcoming, an alternative procedure which provides a compromise between the advantages and drawbacks of hard- and soft-thresholding is proposed. The sigmoid function, also called the sigmoidal curve or logistic function, is defined as:

(7) $$\begin{array}{cc} y=\frac{1}{1+e^{-\beta x}} & \beta \in {\text{R}}^{+} \end{array}$$

The sigmoid function is so-called because it is shaped like one form of the Greek letter sigma. In general, a sigmoid function is real-valued and differentiable, having either a non-negative or non-positive first derivative which is bell shaped. The degree of approximation of the sigmoid function to the signum function can be adjusted by regulating the value of *β*. The sigmoid function only produces an output *y* over the range 0 to +1. By making a simple change of Equation (7) as follows, the output range can be extended from −1 to +1:

(8) $$\begin{array}{cc} y=\frac{2}{1+e^{-\beta x}}-1 & \beta \in {\text{R}}^{+} \end{array}$$

**Figure 3.**
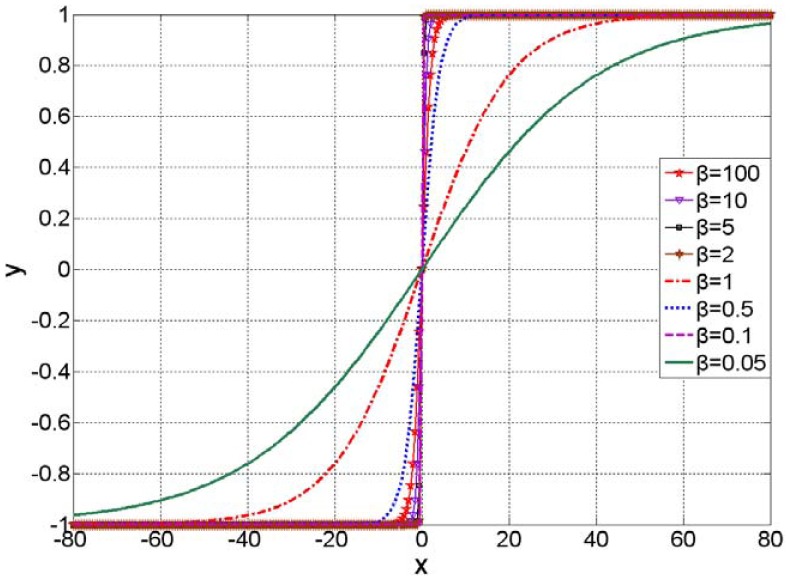
Plot of the modified sigmoid function.

Figure 3 shows the typical plot of the modified sigmoid function with *β* equal to 0.05, 0.1, 0.5, 1, 2, 5, 10 and 100.

Thereby, the modified sigmoid function is very suitable as a thresholding scheme which can be defined as follows:

(9) $${\tilde{\omega }}_{k,j}=\{\begin{array}{ccc} (\omega_{k,j}-T)-\left[\frac{2}{1+e^{\beta \left(\frac{\omega_{k,j}-T}{T}\right)}}-1\right]T, & \text{for}\omega_{k,j}\geq T & \\ 0 & ,\text{for}\left|\omega_{k,j}\right|<T & \beta \in {\text{R}}^{+}\\ (\omega_{k,j}+T)-\left[\frac{2}{1+e^{\beta \left(\frac{\omega_{k,j}+T}{T}\right)}}-1\right]T, & or\omega_{k,j}\leq -T & \end{array}$$

**Figure 4.**
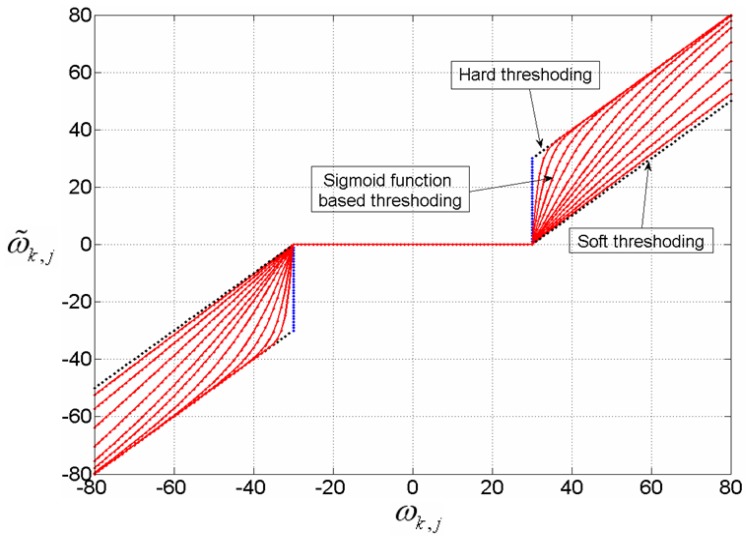
The comparison of soft, hard and sigmoid function based thresholding scheme.

It can be easily proved that the presented thresholding scheme is a kind of compromise between hard and soft thresholding, where the difference is caused by the constant *β*. If *β* = 0, soft thresholding can be considered, and if *β*→ +∞, the equation corresponds to hard thresholding. Figure 4 illustrates the comparison of soft, hard and sigmoid function based thresholding scheme (with *T* = 30 and *β* equal to 0.1, 0.3, 0.6, 1.0, 1.5, 2.0, 3.0, 5.0, 8.0, 15.0 and 30.0).

### 3.4. Threshold Estimation

Although the presented thresholding scheme is simple and effective, selection of a rational threshold value is a crucial task as it directly affects the denoising results. For example, choosing a very large threshold will shrink almost all the coefficients to zero and may result in over smoothing of the measured signals. On the other hand, a small value of threshold will retain the sharp edges and details but may fail to suppress the noise artifacts. Among the existing methods, the most popular one is the universal threshold [15,16]. The universal threshold is defined as:

(10) $$T=\sigma \sqrt{2\ln N}$$

where *N* is the signal length and σ denotes the standard deviation of the noise. The later is estimated from the median of the detail coefficients at the first level of signal decomposition:

(11) $$\sigma =\frac{\left|\text{median}(\omega_{1,j})\right|}{0.6745}$$

The universal threshold may be unwarrantedly large since its dependence on the number of samples. It will yield an overly smoothed estimate and in some cases, the pseudo Gibbs phenomena may appear [17]. Considering that the wavelet coefficients within subbands are, in fact, locally stationary and have dependency both in scale and across scales, here a level-dependent thresholds, which are more adaptive to the noise and signal characteristics, is adopted by the formula:

(12) $$T=\sigma_{i}\sqrt{2\ln N_{i}}=\frac{\left|\text{median}(\omega_{1,j})\right|}{0.6745}\sqrt{2\ln N_{i}}$$

**Figure 5.**
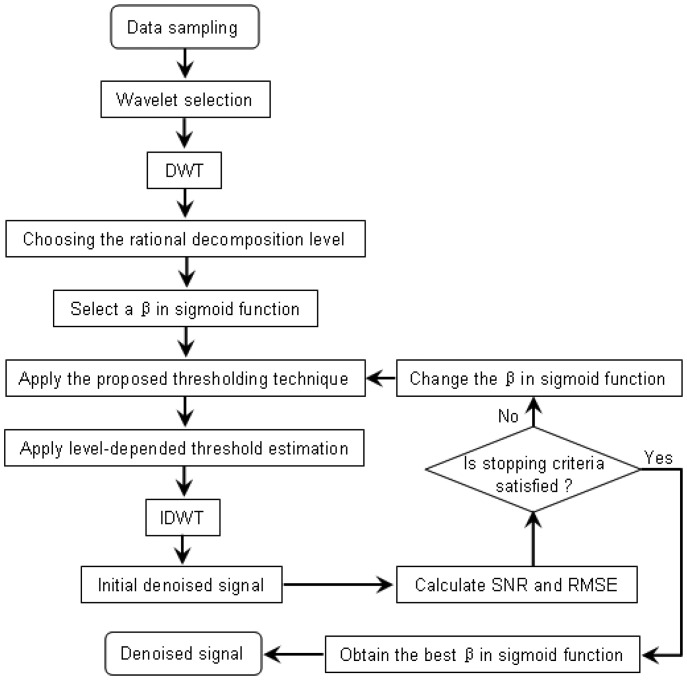
Flowchart of the proposed DWT denoising method.

The proposed denoising procedure is summarized in Figure 5.

## 4. Numerical Simulations and Results

In order to verify the performance of the proposed denoising approach, computer generated noises with variable amplitudes are added to well-known benchmark signals; moreover, the classical algorithms are performed for comparison. A number of quantitative parameters can be used to evaluate the performance of the denoising procedure in terms of the reconstructed signal quality. In this case, the following parameters are compared:

1. Signal-to-noise ratio (SNR):
  (13) $$\text{SNR}(dB)=10\ln\frac{\sum_{k=1}^{N}x^{2}(k)}{\sum_{k=1}^{N}{\left[x(k)-x\prime (k)\right]}^{2}}$$
  where *x′*(*k*) is the denoised signal, and *x*(*k*) is the original signal. The constant, *N*, is the number of samples composing the signals.
2. Root-mean-square error (RMSE):
  (14) $$\text{RMSE}=\sqrt{\frac{\sum_{k=1}^{N}{\left[x(n)-x\prime (n)\right]}^{2}}{N}}$$

### 4.1. Simulation Setting

A simulation experiment is conducted to investigate the performance of the proposed filtering procedures to different signals and to various degradations. For comparison, the simulation involves four known benchmark signals (Block, Bumps, Heavy sine, and Doppler) and three kinds of degrees of degradation. The SNR of the selected signals is 2.0000 dB (heavy blurred case), 5.0000 dB (medium blurred case) and 10.0000 dB (light blurred case) for each benchmark. The computations are performed with the MATLAB for Windows version 7.4 (MathWorks, Natick, MA, USA) [18], using our own developed programs. The MATLAB Toolbox for wavelets 4.0 is used for the library of wavelet filter coefficients. All the simulated signals contain 2,048 samples. The selected signals are transformed into wavelet coefficients by the db4 wavelet, and the rational wavelet decomposition level is 4, which is chosen by the aforementioned “white noise test” method. For the sake of comparison, two existing algorithms, hard- and soft-thresholding approaches, are also implemented here.

### 4.2. Results and Discussion

**Figure 6.**
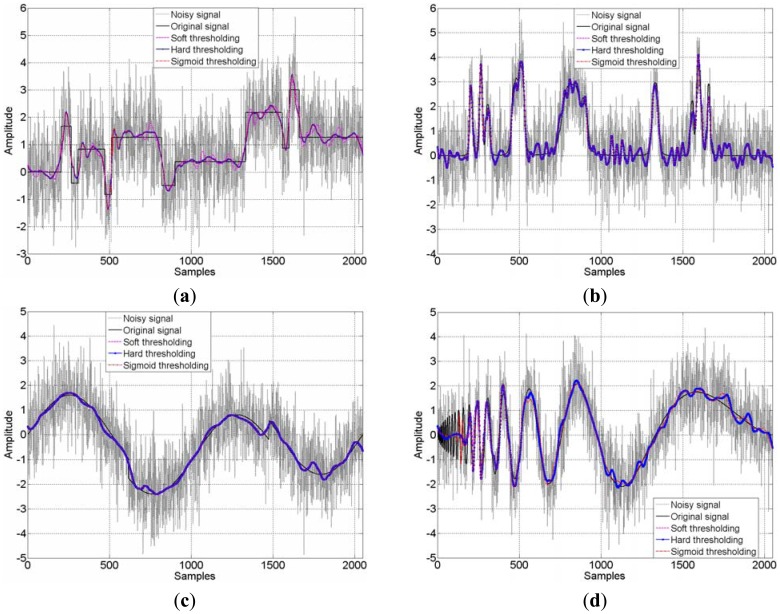
Benchmark signals denoising by db4 wavelet using different thresholding schemes (SNR = 2.0000), (**a**) Blocks signal; (**b**) Bumps signal; (**c**) Heavy sine signal; (**d**) Doppler signal.

**Figure 7.**
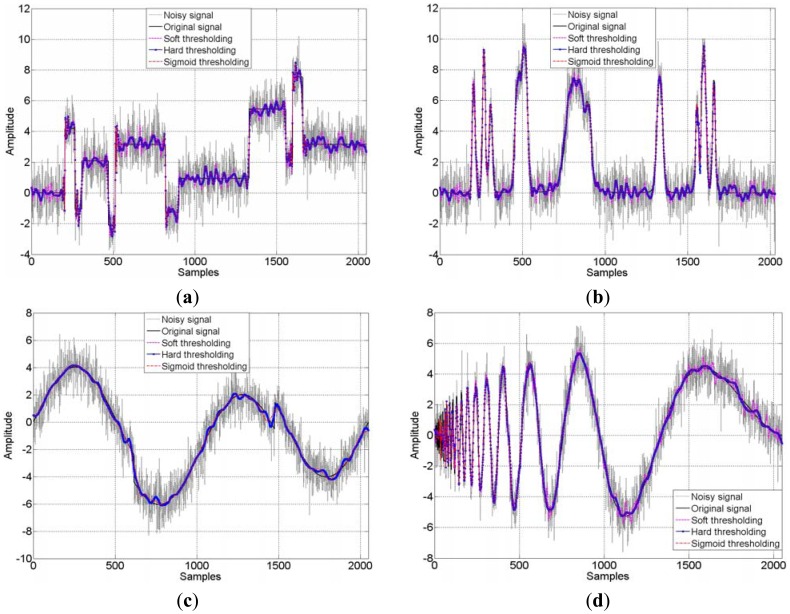
Benchmark signals denoising by db4 wavelet using different thresholding schemes (SNR=10.0000), (**a**) Blocks signal; (**b**) Bumps signal; (**c**) Heavy sine signal; (**d**) Doppler signal.

The detailed results of the three thresholding methods are illustrated in Table 1. As known, sensitivity in the vibration tests could be enhanced by increasing SNR. It can be seen from the table, decrease of the RMSE of residuals and increase of sensitivity are achieved simultaneously. All of the applied thresholding approaches provide at least two-fold improvements in relation to the initial SNR value. One can also see from the comparison, the SNR obtained by using the sigmoid function based thresholding scheme is higher than the hard- and soft-thresholding (at least the same as hard thresholding). This trend is more evident when the light blurred case is considered which reveals that the sigmoid function based thresholding scheme to be an effective filter to some extent, no matter whether the SNR is high or low. The four kinds of noisy signals with the SNR at 2.0000 dB and 10.0000 dB as well as their processed results are displayed in Figures 6 and 7. In the two figures, there are four sub-figures representing the four benchmark signals, respectively. It can be visually appreciated that a great amount of noise has been suppressed. In heavy blurred cases the huge noise is removed and the outline of benchmark signals is recovered; moreover, in light blurred cases the detail of benchmark signals are only slightly harmed after thresholding. Comparing the four sub-figures in Figure 7, one can easily see that the thresholding scheme is very important. The curves obtained by the proposed procedure are more similar to the original signals. This is due to the sigmoid function based thresholding scheme is flexible and adaptive, while the hard- and soft-thresholding method are coarse and rough, hence the proposed method performs the better performance to simulations.

## 5. Shaking Table Test and Data Analysis

The simulation results have shown the good performance of the proposed method for improving the signal and suppressing the noise. In order to verify its ability in an actual vibration experiment, a shaking table test was carried out for this study.

### 5.1. Model Design and Experimental Process

**Figure 8.**
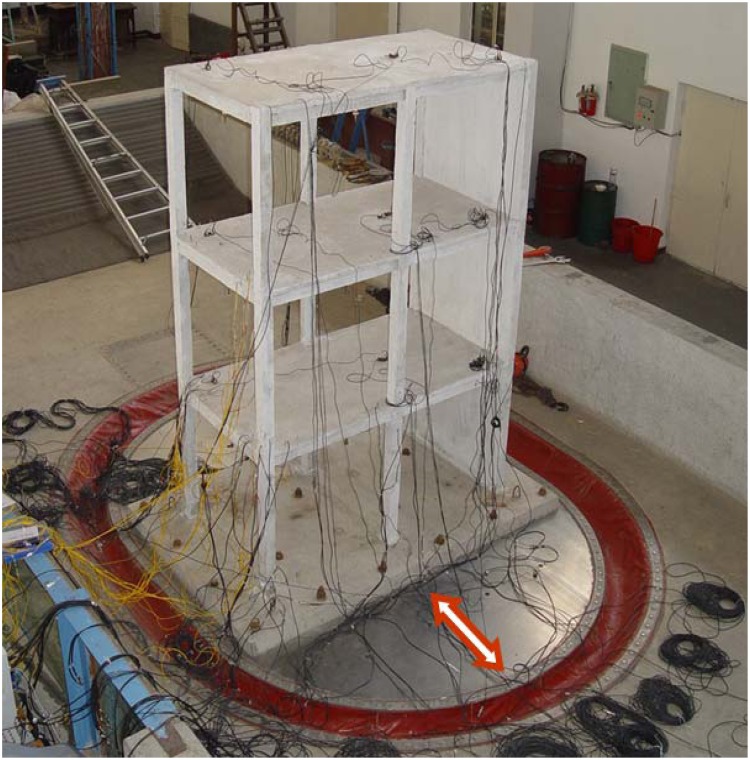
Experimental setup for shaking table tests.

**Figure 9.**
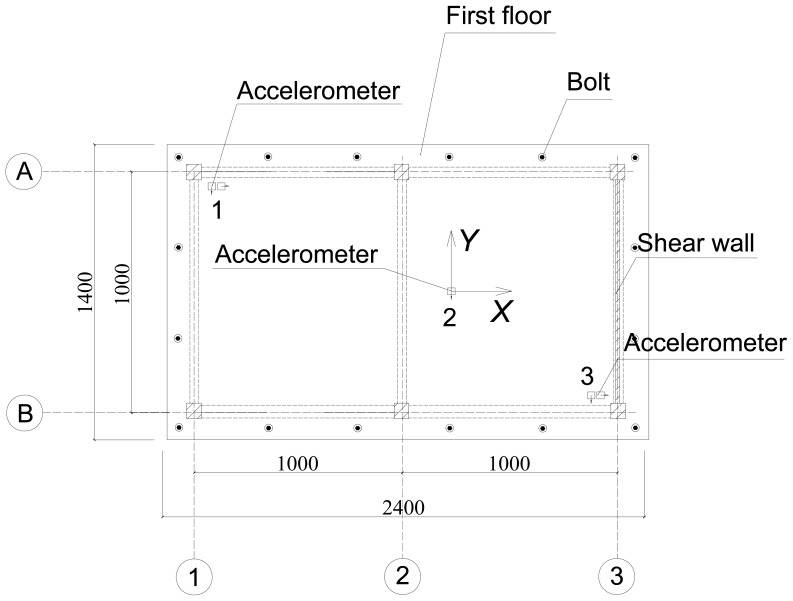
Schematic diagram of accelerator location.

The test was conducted at the State Key Laboratory of Coastal and Offshore Engineering in Dalian University of Technology, China [19]. The tests model the scale of a three-story reinforced concrete frame-shear wall structure with an asymmetric shear wall distribution under simulated seismic excitations, as shown in Figure 8. The height of the structural model is 3.1 m, and the total mass is nearly 3 tons. The cross-section area of the column and beam is 0.08 m × 0.08 m and 0.06 m × 0.1 m, respectively. The thickness of the shear wall is 0.03 m. Accelerometers were installed in each story of the model and the shaking table deck, and they were placed at the center of mass of the structure, and near and away from the sides of the shear wall, respectively (see Figure 9). The base excitations were used by white noise and the data were acquired simultaneously at the rate of 500 Hz from 16 channels.

### 5.2. Real Data Analysis

**Figure 10.**
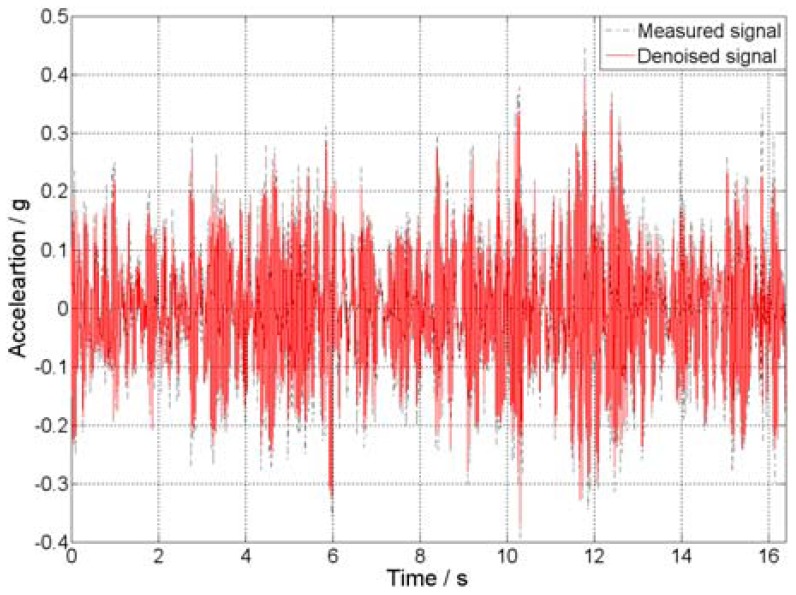
Measured and denoised acceleration response.

The measured and denoised acceleration response at location 2 (center of mass) of every story in the *Y* direction is selected for analyses. Here, the measured signals are also transformed by the db4 wavelet, and the rational wavelet decomposition level is 3, which is chosen by the aforementioned method. Figure 10 shows the measured and denoised acceleration response at the third story of the model. From the visual inspection of the results, it can be observed that the measured signals are corrupted by electromagnetic interference of the environment because the signal receiver has a relative wide bandwidth. The amplitude and arrival time of every noise peak is random, causing a disturbance that masks the objective signal to some extent. After application of the proposed procedure, it can be found that the signal is clearer than the measured signal. Statistical results of before and after filtering is demonstrated in Table 2. It can be seen that the accelerations between −0.39 g and 0.45 g for the measured signal and between −0.37 g and 0.39 g for the denoised signal, which indicates the measured signal after filtering has a smaller spread of coordinates implies that proposed method is effective. In addition, the standard deviations demonstrated in Table 2 are also proved the point.

**Figure 11.**
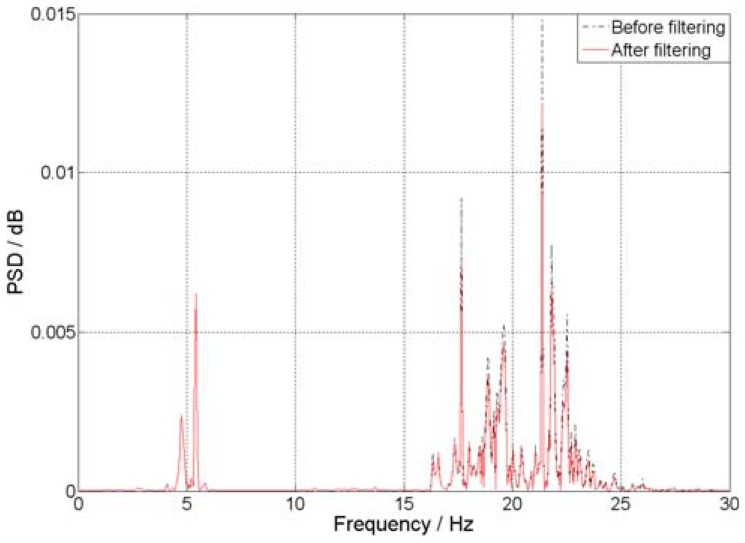
PSD of the signals before and after filtering.

Figure 11 illustrates the power spectrum density (PSD) of signals at the third story of the model before and after processing. It is clear that the noise spectrum is distributed along almost the whole frequency band. Comparing the PSD, one can see the good effect of the proposed method in real signal denoising, even when embedded in broad band noises. From the identified results shown in Table 2, it can be seen, as expected, that the model frequencies are exactly the same. Therefore, although the proposed method works well for the noise, it doesn't destroy the real frequency components of the signal at all.

**Figure 12.**
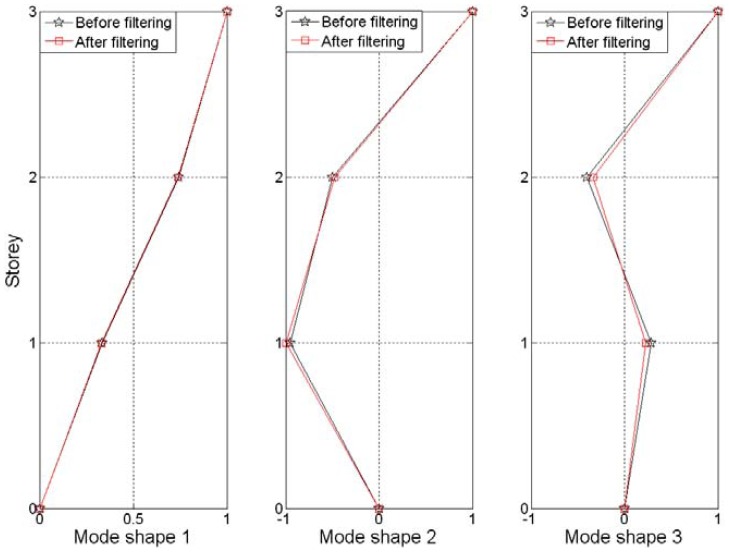
The first three orders of mode shapes of the model.

The first three orders of mode shapes of the model before and after filtering are displayed in Figure 12 and the maximum errors between them are given in Table 2. As can be observed from this data, the first model shapes of the structure are almost the same and the errors become larger with the increases of the mode. It's really an interesting phenomenon that the noise can affect the identification of the mode shape in this trend and the proposed method can achieve a reasonable performance level in reducing noise.

## 6. Conclusions

How to remove the noise interference is a critical issue in vibration experiments. Especially in some tests there are not any prior knowledge of the noise, the conventional filters are not suitable. In the present work, this issue is tackled by a DWT-based denoising approach. To overcome the disadvantages of the traditional hard- and soft-thresholding method, an improved thresholding technique called the sigmoid function-based thresholding scheme is presented. Numerical and experimental results are presented to evaluate the performance of the proposed method, and the results have shown that the method is extremely efficient in eliminating noise, no matter whether the SNR is high or low. The sigmoid function-based thresholding technique can remove small coefficients and shrink large coefficients using its non-linear characteristics to reproduce peaks and discontinuities as accurately as possible, without sacrificing visual smoothness. In short, the proposed method is simple, efficient and could be easily implemented by hardware or software, which is especially suitable in vibration tests.

## Figures and Tables

**Table 1.** Performance evaluation of benchmark signals denoising with different thresholding schemes.

	**Noisy signal**		**Soft thresholding**		**Hard thresholding**		**Sigmoid thresholding**	

	SNR	RMSE	SNR	RMSE	SNR	RMSE	SNR	RMSE
Blocks	2.0000	0.9826	12.8601	0.2814	13.0078	0.2767	13.0926	0.2740
	5.0000	0.9826	14.5781	0.3262	14.7936	0.3182	14.8078	0.3177
	10.0000	0.9826	17.5151	0.4136	17.9304	0.3943	17.9304	0.3943
Bumps	2.0000	0.9826	13.6467	0.2571	13.6467	0.2571	13.6467	0.2571
	5.0000	0.9826	15.9711	0.2779	16.0294	0.2760	16.1072	0.2735
	10.0000	0.9826	19.3004	0.3368	19.6685	0.3228	20.0682	0.3083
Heavy sine	2.0000	0.9826	18.9648	0.1393	18.9648	0.1393	18.9648	0.1393
	5.0000	0.9826	21.1270	0.1535	21.1270	0.1535	21.1270	0.1535
	10.0000	0.9826	23.9296	0.1976	23.9296	0.1976	23.9480	0.1972
Doppler	2.0000	0.9826	12.4548	0.2949	13.0133	0.2765	13.4134	0.2641
	5.0000	0.9826	14.0443	0.3469	14.7786	0.3187	14.8974	0.3144
	10.0000	0.9826	17.7133	0.4043	19.3587	0.3345	19.6503	0.3235

**Table 2.** Experimental results before and after filtering.

	**Acceleration/g**			**Frequency/Hz**			**The max. error of the mode shape/%**		

	**Max.**	**Min.**	**Std.**	**Mode 1**	**Mode 2**	**Mode 3**	**Mode 1**	**Mode 2**	**Mode 3**
Before	0.45	−0.39	0.11	5.43	17.70	21.36	0.36	4.57	6.01
After	0.39	−0.37	0.10	5.43	17.70	21.36			
